# Medical Genetics in Brazil in the 21st Century: A Thriving Specialty and Its Incorporation in Public Health Policies

**DOI:** 10.3390/genes15080973

**Published:** 2024-07-24

**Authors:** Dafne Dain Gandelman Horovitz, Têmis Maria Félix, Victor Evangelista de Faria Ferraz

**Affiliations:** 1 Centro de Genetica Medica, Instituto Nacional de Saude da Mulher, da Criança e do Adolescente Fernandes Figueira, Fundação Oswaldo Cruz, Rio de Janeiro 22250-020, RJ, Brazil; 2 Medical Genetics Service, Hospital de Clinicas de Porto Alegre, Porto Alegre 90035-903, RS, Brazil; tfelix@hcpa.edu.br; 3 Department of Genetics, Ribeirão Preto Medical School, University of São Paulo, Ribeirão Preto 14049-900, SP, Brazil; vferraz@usp.br

**Keywords:** medical genetics, rare disease, genetic, Brazil, public health policies

## Abstract

Brazil is a continent-size country with 203 million inhabitants, classified as a developing upper-middle-income country, although inequities remain significant. Most of the population is assisted by the public Unified Health System (SUS), along with a thriving private health sector. Congenital malformations are the second leading cause of infant mortality and chronic/genetic disorders and a significant burden in hospital admissions. The past two decades have been crucial for formalizing medical genetics as a recognized medical specialty in the SUS, as well as for implementing a new health policy by the Ministry of Health for comprehensive care for rare diseases. These public health policies had the broad support of the Brazilian Society of Medical Genetics and Genomics and patient organizations. Most comprehensive genetic services are concentrated in large urban centers in the South and Southeast regions of Brazil; with this new policy, new services throughout the country are progressively being integrated. The number of medical geneticists increased by 103% in a decade. Details on the policy and an overview of the availability of services, testing, human resources, newborn screening, research projects, patient organizations, and relevant issues regarding medical genetics in this vast and diverse country are presented.

## 1. Introduction

Medical practice in genetics began in Brazil in the 1950s, with Brazilian scientists shifting their work to this new emerging field of research following a worldwide tendency [1,2,3]. The Brazilian Society of Genetics was founded in 1955 [4], bringing together human biologists and physicians. Due to the necessity to establish the specialty of medical genetics in the country in 1986, the Brazilian Society of Clinical Genetics (currently named the Brazilian Society of Medical Genetics and Genomics) was created [5,6]. Brazil is a continent-size country with a 203 million population [7], the largest and most populous in South America. It is classified as a developing upper-middle-income country, although inequality remains significant. The public Unified Health System (“Sistema Único de Saúde” or SUS) was created in 1990 by law 8080/90 [8] and is one of the largest and most complex public health systems in the world, ranging from simple care to organ transplantation, guaranteeing full, universal access and free for the entire population in the country.

In addition to SUS, 28.5% of the Brazilian population has some sort of private health insurance coverage. This percentage is much higher in urban than in rural areas and higher in the Southeast/South regions [9]. Insurance companies are regulated by a National Supplementary Health Agency (ANS), a federal government organization established in 2000 that periodically issues guidelines for coverage of medical/surgical/laboratory procedures, including diagnosis in genetics [10]. There is a thriving private health sector in the country, with medical care and access to complex investigation like most developed countries.

The country has undergone an epidemiological transition in recent decades, where congenital malformations shifted to the second leading cause of infant mortality since the year 2000 [1,11], with control of deaths due to infectious, respiratory diseases, malnutrition and others, placing only behind perinatal disorders, the first cause of mortality in children one year and under. Regarding congenital malformations in the Brazilian Ministry of Health’s database, the 2023 hospital mortality rate in the pediatric age range (0 to 19 years) was 2.5 times greater when the primary diagnosis for admission was included in chapter XVII of ICD-10. An increment of the permanency and hospitalization cost for this group of diseases can also be noticed [12].

In a world with the steady improvement of health, there has been a global decline in disability-adjusted life-years (DALYs); such rate, however, has shown stagnation, mostly related to a shift towards a greater proportion of burden due to years lived with disability (YLDs) [13], a trend that is also being observed in Brazil. The demand for health services to deal with disabilities has been increasing.

Brazil still faces multiple obstacles to improving and expanding genetic services due to its wide territory, sociocultural inequalities, and major basic health problems. Genetic services are mostly concentrated in large urban centers in the South and Southeast regions, primarily in tertiary care university hospitals. Demand for genetic services has been increasing, especially with the aforementioned epidemiological transition. From 2004 on, government initiatives and, later, health policies began to be elaborated, which have led to medical genetics being officially recognized as important for the diagnosis and care of rare genetic disorders. This article will focus on multiple topics related to the practice of medical genetics in the country: epidemiology, service provisions, genetic testing and counseling, patient registries, patient empowerment, access to clinical trials and new therapies, and local policies related to genetic/rare diseases.

## 2. A Health Policy for Genetic/Rare Diseases in Brazil

The Brazilian Ministry of Health published a decree in 2009 that proposed the creation of a “National Policy for Comprehensive Care in Clinical Genetics at SUS” (Política Nacional de Atenção Integral em Genética Clínica no SUS) [5,14]. The process that led to acknowledging the need to establish such a policy began in 2001 and was partly influenced by the announcement of the sequencing of the human genome. At the time, a committee to study and regulate the use of the human genome was formed. As initiatives regarding care in genetics far transcended the initial attributions of the committee and depended on a specific action of the Ministry of Health, a Working Group (WG) was formally established in 2004 [15], aiming to assess the situation of care in clinical genetics and to promote a debate on the various instances of the SUS, involving local health care managers and service providers, as well as to obtain grants for the development of the policy.

Despite its publication in January 2009, the National Policy for Comprehensive Care in Clinical Genetics within the SUS [14] was never actually implemented, as a supplementary ordinance, with specific directives and financing not in place. Nevertheless, supported by the epidemiological transition, the need for organized action in the area of medical genetics/birth defects in Brazil had been acknowledged. 

Finally, in 2012, a new WG, at that moment not as a technical demand but pushed forward by patient organizations, was created to elaborate a policy directed to rare diseases (RD). This shift, leading to user demands and participation, working in collaboration with a technical team summoned by the Ministry of Health, led to, in 2014, the institution of the National Policy for Comprehensive Care to People with Rare Diseases (NPCCPRD) at SUS [16].

The main goal of the NPCCPRD is to reduce morbidity, mortality and secondary manifestations, and to improve the quality of life of the patients through promotion, prevention, and early detection activities, preventing disability, and promoting palliative care. To achieve the intended goals, rare disease centers needed to be established or formalized as such. The policy is directed for rare diseases, both genetic and non-genetic, being each axle subdivided into care lines (genetic origin: birth defects and late-onset disorders, intellectual disabilities and inborn errors of metabolism; non-genetic origin: infectious diseases, inflammatory, auto-immune, and other non-genetic rare disorders) [16]. Reimbursement is patient-centered, depending on the specific care line needed, and a fixed value is intended to cover clinical evaluation and investigation, including specific diagnostic genetic tests as needed (from karyotype to next-generation sequencing).

The launch of such a policy was a landmark for the recognition of the importance of medical genetics in the SUS, although the implementation of reference services has been slow and does not include several units that are also capable of offering diagnosis and care for genetic disorders. Nevertheless, the NPCCPRD has been acting as an induction factor for the formalization, creation, and organization of services throughout the country, where having a clinical geneticist and the possibility of ordering diagnostic exams are mandatory.

**Figure 1:**
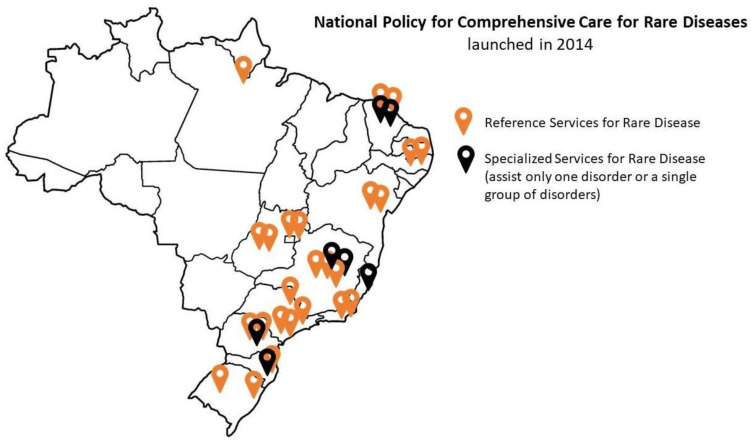
Map of Brazil showing the location of 32 services for rare diseases formally included in the network according to the National Policy for Comprehensive Care for Rare Diseases (updated June 2024).

Up to early June 2024, ten years after the publication of NPCCPRD, there were 32 services formally licensed, most of these offering comprehensive care for genetic disorders (Figure 1) [17]. More than 133,000 consultations have been offered to RD patients. Hopefully, more centers will join the network in the near future, as the habilitation process has been running more smoothly in the past five years. In 2023, an RD General Coordination, linked to the Specialized Attention Department of the Ministry of Health, was created. Recently, an ordinance was published by the Ministry of Health, establishing a technical group including members of the Ministry of Health, medical societies, and geneticists to follow up and render the policy more effective [18].

## 3. Medical Genetics in Brazil: Genetic Services Provisions, Genetic Testing, Clinical Genetics, Genetic Counseling

As previously stated, medical practice in genetics in Brazil has had a recent onset when compared to other medical specialties. Genetic services began in research settings and universities (where many still exist), and only recently robust services with care in genetics became available in the public health system. Most genetic services in teaching hospitals and academic settings are also usually linked to SUS. With the introduction of the NPCCPRD new services were implemented, reinforcing the inductive nature of such policy in enhancing the offer of consultations for rare, but especially for genetic diseases [15,19]. 

**Figure 2:**
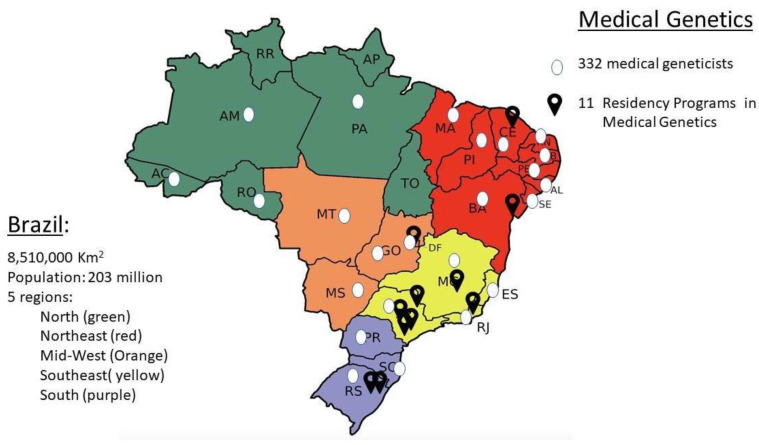
Map of Brazil, showing the five regions of the country, the federative states, and the distribution of medical geneticists across the country (white dots) and residency programs in medical genetics (black arrow).

One important factor for access to specialized care is the availability of a working force. According to Bonilla et al., in 2020, there were 332 medical geneticists in Brazil, 208 of whom had completed a residency program in medical genetics. Of all medical geneticists, 25% also had a degree in pediatrics. The specialists in genetics were active in 23 of the 27 federal units of Brazil (which comprises 26 states plus the Federal District), although highly concentrated in the state capitals or in the most populated cities and in the Southeastern and South regions of the country (77% of the workforce) (Figure 2). These two regions are the wealthiest and the most populated in Brazil. On the other hand, the northern region, which comprises seven states in the Amazon area, with a population of around 18 million, is served by only five medical geneticists [20].

The first medical residency program in medical genetics was established at Ribeirão Preto Clinical Hospital in 1977, and presently, 11 medical residency programs are available for training in medical genetics, which leads to around 25 new specialists/year [20,21]. Almost all programs are in the Southeast/South regions, except for one in the Midwest in the Federal District and two in the Northeast (Figure 2). This follows the distribution of the physicians and services, and is ultimately one of the pitfalls for spreading care in medical genetics in the more underserved regions of Brazil. 

Despite the insufficient number of medical geneticists in Brazil, interesting data are shown in a publication by the Brazilian Medical Association [22]. The aforementioned demonstrates that the number of geneticists increased by around 103% between 2012 and 2022, and the number of first-year medical residents in medical genetics increased by 53% between 2018 and 2021, being the third medical specialty that grew the most in the period.

The quality of the services available, from a clinical standpoint, is compatible with clinical genetic services around the world, including those from Europe and North America. Most pitfalls, however, are related to the limited availability of diagnostic testing in the public health sector, although the situation has been changing especially within the past decade [5,23]. On the other hand, genetic services in private medical offices and clinics are available in the main cities in Brazil. Their distribution follows the same rationale of the general distribution of the public genetic services. What differentiates private care is easier access to a first consultation and diagnostic exams, including the most sophisticated, such as next-generation and exome sequencing, which have mandatory coverage by private insurance according to specific guidelines [10]. The private sector comprises a robust laboratory network, which offers most types of genetic testing. 

Genetic counseling in Brazil is delivered predominantly by medical geneticists, with a few exceptions (such as cancer centers dealing with inherited cancer, primary care units and the delivery of information regarding sickle cell trait after newborn screening, for instance). The profession of genetic counselor is not recognized in Brazil, although genetic counseling is specifically listed and reimbursed by the NPCCPRD, and non-physicians can participate in multidisciplinary teams in the services [15].

Reproductive genetics, including pre-implantation genetic testing and prenatal diagnosis/screening, are available, although mostly to a very privileged minority of the population who can afford to pay out of pocket. Some university/teaching hospitals provide prenatal genetic counseling and limited prenatal genetic diagnosis. Abortion is not legal in Brazil, with few exceptions: in cases of pregnancy resulting from rape or if the continuation of the pregnancy is considered life-threatening for the mother (Brazilian Criminal Code of 1940), and more recently if the fetus has anencephaly [24]. Nevertheless, when facing unfavorable diagnoses, many pregnant women search for illegal pregnancy termination. Unfortunately, this is only easier and safer to access to the most privileged. In cases of very severe fetal malformations and when a diagnosis is timely, some women appeal to legal authorization for termination, which may be granted on an individual basis, depending on each judge´s decision. 

## 4. Newborn Screening

The National Newborn Screening Program (*Programa Nacional de Triagem Neonatal*—PNTN) was established by the Ministry of Health in 2001 [25]. There have been some updates over the years, with the inclusion of more diseases. In 2014, screening was recommended for six diseases, beginning with congenital hypothyroidism (CH) and phenylketonuria (PKU), progressively incorporating more diseases (sickle cell disease and other hemoglobinopathies (SC), cystic fibrosis (CF), congenital adrenal hyperplasia (CAH) and biotinidase deficiency (BD)), according to each state´s program capacity. In 2021, the PNTN was once again updated, not only due to public demands, especially from families affected by inborn errors of metabolism, but also due to better and cheaper technology available. The present program now considers the six diseases previously cited, plus congenital toxoplasmosis, as phase I. The other incorporated diseases to be screened for are phase II—galactosemia, amino acid disorders, urea cycle, and fatty acid oxidation disorders; phase III—lysosomal storage disorders; phase IV—primary immune deficiencies; phase V—spinal muscular atrophy. The incorporation of phases II through V also varies among states and depends on installed capacity. Presently, very few states offer complete testing. Newborn screening coverage in 2020 was 82.53% [26].

## 5. Treatment of Genetic/Rare Diseases

New (and high-cost) treatments are being developed and have been increasingly available for genetic diseases, such as for several lysosomal storage disorders, growth hormone for Turner and Prader Willi Syndromes, and modulators for cystic fibrosis, among others. In 2017, the registration of medications for RD was standardized with expedited procedures in Brazil with recommendations to approve clinical trials, recommendations for good manufacturing practices, and the registration of new medications for rare diseases [27]. Up to January 2024, 116 medications have been commercially approved; however, in Brazil, when patients and families try to obtain new drugs as soon as they become available, they usually undergo lawsuits against the government based on the constitutional right that states that all citizens have the right to health, to be provided by the state. Treatment requests made by families and patient associations, based on the Federal Constitution, are almost always abided by courts. Such court decisions will lead to individual and unplanned drug purchases, leading not only to excessive costs but eventually to incorrect management of diseases. 

To enhance access, rationalize, and render therapies more efficient, clinical protocols and therapeutic guidelines (Protocolos Clinicos e Diretrizes Terapeuticas—PCDT), organized by the Ministry of Health with the participation of invited specialists in each disease have been establishing criteria for diagnosis of genetic/rare diseases, as well as recommending treatments with specific medications and other appropriate products when applicable. Such guidelines must be based on scientific evidence, considering efficacy, safety, effectiveness and cost-effectiveness criteria, and have been very important to render access to treatment for patients with genetic/rare diseases. A recent presentation of ANVISA during an open audience about rare diseases at the Federal Senate in February of 2024 showed that, until June of 2023, 122 products (5 gene therapies, 49 biological products, and 68 specific medications) were approved for use in Brazil [28]. The Ministry of Health states that it has incorporated 96 medications used exclusively to treat 54 RD in the SUS. The financing of 66 of these exclusive medicines is under the responsibility of the Ministry of Health itself, while the financing of 26 is the responsibility of each federative state and the Federal District [29]. A list of some clinical protocols and guidelines related to rare and genetic diseases approved in the Public Health System is in Table 1, which include from special milk formulas to gene therapy.

Despite all these initiatives, the impact on rare disease care is modest. A recent study described the access to medication for RD in Brazil [30] and highlighted the growing numbers, between 2014 and 2020, of clinical trials, recommendations for incorporating medications, new clinical protocols, and therapeutic directives. Despite these efforts there is poor alignment with care policies, pricing methods, technological development, or process management in pharmaceutical assistance, lacking articulation in the care network. 

It should also be noted that, along with the treatment programs, strategies for the prevention of new cases need to be outlined, being the role of medical genetics and genetic counseling essential for the process. Genetic counseling is one of the few possibilities for stabilizing the treatments’ rising costs [31].

## 6. Genetic Epidemiology

Despite the RD Policy of 2014, there is a lack of epidemiological data on RD in Brazil. The existing data so far are restricted to a few disorders. The newborn screening program provides data on six selective disorders [32]. Another governmental program is the Brazilian Live Birth Information System (SINASC), which registers all newborns and mandatory description of eight congenital anomalies (neural tube defect, microcephaly, congenital heart disease, orofacial clefting, genital anomalies, limb defects, abdominal wall defect, and Down syndrome) is required [33]. However, from 2010 to 2021, 34,559,375 newborns were registered, and 2,852,956 (0.83%) presented a congenital anomaly, showing that congenital anomalies are still under-reported. The majority (83%) were isolated anomalies. Data showed a higher rate of registration of congenital anomalies in 2016, the time of the Zika virus epidemic causing microcephaly [34].

Another ongoing registry specific for craniofacial anomalies is the Brazilian Craniofacial Project [35]. This initiative collects data on craniofacial anomalies in ten healthcare centers using a standard protocol and provides genetic testing for craniofacial disorders, such as analysis for 22q deletion and, more recently, exome sequencing (ES) [36].

To provide epidemiological data on RD in Brazil, the Ministry of Health through the National Council for Scientific and Technological Development (Conselho Nacional de Desenvolvimento Científico e Tecnológico; CNPq) financed an initiative of RD survey, the Brazilian Rare Disease Network (RARAS). The main objective of this study is to perform an inquiry into epidemiology, clinical findings, diagnostic and therapeutic resources, and costs of RD in Brazil. This study includes 40 healthcare centers located in all five regions of Brazil. It has two phases: a retrospective phase, when all cases assisted at the centers from 2018 to 2019 were reviewed, and an ongoing prospective phase [32].

One aim of the Brazilian Rare Disease Network was to study the availability of genetic tests at centers provided by SUS. Data showed that despite the inclusion of many genetic tests at the NPCCPRD, many centers do not offer most of these tests [23]. Many genetic tests are outsourced to public services through diagnosis networks that provide mainly inborn errors of metabolism laboratory tests [37].

Two national institutes funded by CNPq related to genetics and RD have been in place in Brazil. The first one, funded in 2009, is the Population and Medical Genetics National Institute (from Portuguese—Instituto Nacional de Genética Médica Populacional—INAGEMP) that has two main projects: Latin American Collaborative Study of Congenital Malformations (ECLAMC) with the aim to perform congenital anomalies surveillance in Latin America and National Census of Brazilian populational groups with high frequency of genetic diseases (CENISO) [38]. More recently, in December 2023, a second initiative, Rare Disease National Institute (InRaras), has been in place with the aim to collect data on the epidemiology of RD and provide ES to selected undiagnosed cases [39].

## 7. Integrating Genomic Medicine in the Health System

In 2020, the Ministry of Health launched the National Program of Genomic and Precision Health–GENOMAS BRASIL [40], a Science, Technology, and Innovation program with the aim to structure the basis to implement precision medicine in the SUS. The program aims to encourage national scientific and technological development in the areas of genomics and precision health, to promote the development of the national genomic industry, and to establish proof of concept for a line of care in genomics and precision health within the scope of the SUS. By 2023, more than 80 projects have been financed [41]. Implementation of genomic medicine for rare diseases is the objective of two initiatives: the Rare Genomes Project (GENOMAS RAROS) and the Cardiovascular Genome Project (RENOMICA). Rare Genomes Project has been using Whole Genome Sequencing (WGS) to diagnose RD from 21 different centers in Brazil. The goal is to sequence 9000 individuals [42]. RENOMICA uses exome sequencing (ES) to diagnose hereditary cardiovascular disorders of 2400 individuals across the country and provide evidence of the cost-effectiveness of genetic tests for cardiovascular disorders [43]. 

Two other programs that are part of the GENOMAS BRASIL initiative are worth highlighting. The ONCOGENOMAS BRASIL project is sequencing genomes of 300 patients with breast cancer and 250 with prostate cancer [44]. Another project recently approved is GENOMA SUS, which aims to capture the genome of 21 thousand Brazilians, focusing on infectious, heart, and brain diseases, among others of great impact on the SUS [45].

Despite these various initiatives to generate genomic data on the Brazilian population, there has been limited impact so far in health care within the scope of the SUS. Structuration of comprehensive care, with qualified detection in the health network at all levels, including patient referral to specialized care services, the provision of genetic counseling, the availability of appropriate genetic tests, multidisciplinary management, and the provision of appropriate treatment medications, continue to be a challenge.

## 8. Patient Organizations

Patient associations have played an increasingly important role in the scenario of genetics and RD in Brazil. These groups became progressively more organized from the 1990s onwards and played an important role in the construction and establishment of the NPCCPRD in 2014 [46]. Patient organizations have also been quite active in collaborating with clinical research sites, helping with information, and even recruiting potential participants. In the past 15–20 years several international collaborative clinical trials for RD have taken place in Brazil, including some studies for the development of new orphan drugs. This has been providing patients with an opportunity to access experimental therapies [47].

Currently, several associations are organized into federations, such as FEBRARARAS, with the aim of achieving greater representation in the scenario, focused on inducing and establishing national healthcare policies [48]. Even initiatives directly linked to care have appeared in Brazil in recent years. Casa dos Raros is an initiative of two civil society organizations focused on the area of RD to establish a comprehensive and multidisciplinary assistance network for people with RD. The first unit is already in operation in Porto Alegre, a city in the South of Brazil [49].

## 9. Conclusions

This century brought rapidly evolving technology, with amazing developments in genetics, including more affordable/cost-effective investigational resources and the development of new treatment frontiers for RD. Such technology has had growing availability in the country, hopefully unfolding in better patient care. 

The past 20 years have been very active for Medical Genetics in Brazil. Medical genetics is one of the growing medical specialties in Brazil; however, an unequal distribution of physicians across the country must be corrected. Working beyond university hospitals and reference institutions, directly and continuously in contact with the Ministry of Health and with policymakers, the specialty has finally been recognized as such in the Unified Health System. The Brazilian Society of Medical Genetics and Genomics and patient organizations were essential players in this process. This has led to more services throughout the country and more options for the population. The constitution of a special policy for comprehensive care in RD, with specific financing strategies, induced and with its implementation and consolidation followed by the Ministry of Health, is finally leading to better access to diagnosis and care of the population. 

Although there is still much to be accomplished, extensive progress has been achieved, and medical genetics has been recognized to be relevant and necessary for the well-being of the population. Not all aspects of this medical specialty in the country, however, have been discussed, and surely dealing with a rare disease policy goes beyond medical genetics alone. It is also very important to note that economic impact and quality of life studies are essential to demonstrate the efficacy of such policies or to point toward different directions.

Even with all the development of recent decades, the limitation of the impact on the Brazilian population is due to some knots that must be untied: the restricted number of specialized services in the various regions of the country, better planning of the referral and counter-referral system, underfunding, and the number and qualification of human resources. These are challenges to be faced in the coming years for the necessary comprehensive health care of those with genetic diseases in Brazil.

## Figures and Tables

**Table 1:** Clinical Protocols and Guidelines related to genetic and rare diseases approved and under application for the Brazilian Public Health System.

Acromegaly
Aggressive Behavior in Autism Spectrum Disorder
Amyotrophic lateral sclerosis
Angioedema associated with C1 esterase deficiency
Biotinidase Deficiency
Classic homocystinuria
Congenital Adrenal Hyperplasia
Congenital Hypothyroidism
Cystic fibrosis
Epidermolysis Bullosa
Fabry disease
Familial Amyloidotic Polyneuropathy
Gaucher disease
Growth Hormone Deficiency–Hypopituitarism
Hemophilia A—Use of Emicizumab
Hereditary Ichthyosis
Infantile Hemangioma
Intellectual Disability
Mucopolysaccharidosis type I
Mucopolysaccharidosis type II
Mucopolysaccharidosis Type IV A
Mucopolysaccharidosis Type VI
Mucopolysaccharidosis Type VII
Neuronal Ceroid Lipofuscinosis type 2
Niemann-Pick Disease Type C
Osteogenesis Imperfecta
Phenylketonuria
Pompe disease
Primary Immunodeficiency with Predominant Antibody Defects–Human Immunoglobulin
Sickle Cell Disease
Spinal Muscular Atrophy 5q Types 1 and 2
Turner syndrome
Wilson’s disease

## Data Availability

The original contributions presented in the study are included in the article, further inquiries can be directed to the corresponding author.
